# Understanding, perceptions and self-use of complementary and alternative medicine (CAM) among Malaysian pharmacy students

**DOI:** 10.1186/1472-6882-11-95

**Published:** 2011-10-13

**Authors:** Syed S Hasan, Chew S Yong, Muneer G Babar, Cho M Naing, Abdul Hameed, Mirza R Baig, Shahid M Iqbal, Therese Kairuz

**Affiliations:** 1 School of Pharmacy, The University of Queensland, 20 Cornwall Street, Woolloongabba, 4102, Brisbane, Australia; 2 International Medical University, Jalan Jalil Perkasa 19, Bukit Jalil, Kuala Lumpur, 57000, Malaysia; 3 Faculty of Pharmacy, University Technology Mara, Bandar Puncak Alam, 42300, Selangor, Malaysia; 4 Faculty of Pharmacy, AIMST University, Jalan Bedong, Semeling, 08100, Bedong, Kedah, Malaysia

## Abstract

**Background:**

In recent times the basic understanding, perceptions and CAM use among undergraduate health sciences students have become a topic of interest. This study was aimed to investigate the understanding, perceptions and self-use of CAM among pharmacy students in Malaysia.

**Methods:**

This cross-sectional study was conducted on 500 systematically sampled pharmacy students from two private and one public university. A validated, self-administered questionnaire comprised of seven sections was used to gather the data. A systematic sampling was applied to recruit the students. Both descriptive and inferential statistics were applied using SPSS^® ^version 18.

**Results:**

Overall, the students tend to disagree that complementary therapies (CM) are a threat to public health (mean score = 3.6) and agreed that CMs include ideas and methods from which conventional medicine could benefit (mean score = 4.7). More than half (57.8%) of the participants were currently using CAM while 77.6% had used it previously. Among the current CAM modalities used by the students, CM (21.9%) was found to be the most frequently used CAM followed by Traditional Chinese Medicine (TCM) (21%). Most of the students (74.8%) believed that lack of scientific evidence is one of the most important barriers obstructing them to use CAM. More than half of the students perceived TCM (62.8%) and music therapy (53.8%) to be effective. Majority of them (69.3%) asserted that CAM knowledge is necessary to be a well-rounded professional.

**Conclusions:**

This study reveals a high-percentage of pharmacy students who were using or had previously used at least one type of CAM. Students of higher professional years tend to agree that CMs include ideas and methods from which conventional medicine could benefit.

## Background

National Center of Complementary and Alternative Medicine (NCCAM) defined complementary and alternative medicine (CAM) as a group of diverse medical and health care systems, practices, and products that are not presently considered to be part of conventional medicine [1]. Due to the increasing demand from the public for more information regarding CAM, the understanding, perceptions and self-use of CAM among undergraduate health sciences students have become a topic of interest. This creates a challenge for the training of future pharmacists in order to gain adequate knowledge to recommend and counsel on CAM. Deeper understanding and acceptable perceptions about CAM among pharmacy students will be fundamental in developing a professional image as providers and advisors on conventional medicines and CAM.

In Malaysia, non-conventional medicines are usually referred to as Traditional and Complementary Medicine (T&CM) and are classified into six main types, namely Traditional Malay Medicine (TMM), Traditional Chinese Medicine (TCM), Traditional Indian Medicine (TIM), Complementary Medicine (CM), Homeopathy and, recently included, Islamic Medical Practice (IMP) [2,3]. In the year 2001, the Malaysian government established National Policy on T&CM with the vision of integrating the use of these non-conventional medicines into the Malaysian healthcare system. The aim of the policy was to ensure quality and safe use of T&CM practices and products to attain optimal potential in healthcare delivery [4].

In view of the recent upsurge of interest in CAM, a number of studies about the understanding, perceptions and self-use of CAM amongst students undertaking healthcare professional courses have been conducted in different countries [5-10]. However, limited data on CAM has been published related to CAM use in Malaysia, and although legislation about T&CM has been introduced, formal incorporation of CAM into healthcare curricula has yet to be established. Most of the studies involved medical students and to a lesser extent, pharmacy students. Among the studies reported, Australia has the highest percentage (93.7% and 78%) of pharmacy students who used CAM [6,7] followed by students in Hong Kong (38%) and United Kingdom (UK) (43%) [8,9]. Generally, most of the studies reported positive attitudes towards CAM among a large proportion of undergraduates and a desire to include CAM education in their academic studies. For instance, in the United States (US) the majority of students surveyed (83%) supported the integration of CAM into their curricula [10]. A Czech study revealed that 90% of the first and third year Czech pharmacy students would agree to recommend CAM to the patients during the consultations concerning healthcare [11].

Medical students in Canada were exposed to less education about CAM than their pharmacy student colleagues and viewed CAM as less useful in their future careers [12]. On the contrary, a study in the US reported that medical students indicated more positive attitudes towards CAM than pharmacy and nursing students [13]. Moreover, Australian pharmacists (91% of 484 respondents) also concurred that it is essential for them to possess knowledge on both conventional and complementary medicines [14]. The aims of the current study were to assess the understanding and perceptions of CAM among pharmacy students in Malaysia, and their self-use of CAM.

## Methods

### Study design and population

This cross-sectional study was carried out among first to final-year (4^th ^year) undergraduate pharmacy students using a self-administered questionnaire. In order to gain a general picture of understanding, perceptions and self-use of CAM among pharmacy students, both public and private university students were included in this study. The private universities that participated were International Medical University (IMU) and Asian Institute of Medicine, Science and Technology (AIMST) and the public University Technology Mara (UiTM). One staff member from each university coordinated the distribution and collection of questionnaires which were anonymous. The study was approved by the International Medical University's research and ethics committee.

### Development of questionnaire

The questionnaire was developed after a detailed review of relevant literature [5-7,15-19]. In addition, some novel questions were developed in accordance with the study objectives. The questionnaire consisted of seven sections (Table 1) with a total of 35 questions.

**Table 1:** Sections divided and the respective type of question

Section	Type of Question
A	Demographics and socio-economical information
B	Barriers to CAM use
C	Sources of information
D	Understanding about CAM
E	Self-practice or use of CAM
F	Perceptions about CAM
G	Integration of CAM into curriculum

Ten questions (section D) evaluated the understanding of CAM using a Likert scale of one to seven reflecting strongly disagree to strongly agree. Students' perception towards the impact of CAM (Section F) used a scale of one (very effective) to five (very harmful). Barriers to the use of CAM (Section B) were explored by providing options and participants could select more than one option. A 5-point Likert scale (strongly agree to strongly disagree) was used to assess opinions about the integration of CAM into the co-curriculum of pharmacy course (Section G). 'Current CAM use' was defined as the use of at least one type of CAM at the time of completing the questionnaire and 'previous CAM use' was defined as the use of at least one type of CAM at by time in past. The questionnaire was worded in English language and was not translated to other languages as English is the medium of instruction at both public and private universities in Malaysia. For the purpose of survey, "CAM was referred to practices, approaches, knowledge and beliefs incorporating plant, animal and mineral based medicines, spiritual therapies, manual techniques and exercises" (WHO 2003).

### Validation of questionnaire

The content of the questionnaire was piloted among ten senior lecturers in the faculty of pharmacy at International Medical University (IMU) and their feedback was incorporated into the revised questionnaire. In the second phase of piloting, twenty pharmacy students from different cohorts were selected to complete the questionnaire on two different occasions fifteen days apart. Through identifying the similarities and differences in response at each occasion, the validity of the question was established; no significant differences were found during this phase. Furthermore, reliability of the questionnaire was assessed using Cronbach-alpha test and the value was found to be 0.78 which was considered reliable.

### Estimated sample size

Sample size was calculated based on three factors; response rate (P), margin of error (D) and Q (1-P). Based on the calculation with response rate 50% and margin of error 5%, the total sample size required for this study was 369 students. However at the end of the data collection phase, 505 students participated in this study which was more than the calculated sample size of 369. Five questionnaires were excluded from the analysis due to incomplete information.

### Sampling

During the data collection phase, one of the researchers approached each cohort of students at IMU to provide information about the study and distribute the questionnaires to the students. Questionnaires were posted via courier service to the coordinator at the other two universities, together with a copy of the ethical approval letter, participant information sheets and consent forms. Sampling of students from the target population occurred in the following manner: every second student on the alphabetical class list was invited to complete the questionnaire. These students were given a reasonable period of time to complete the questionnaire. The detailed of the students population size and number of students participated in the study is presented in Table 2.

**Table 2:** Eligible population size and students participated in this study

Year of study	UiTM		AIMST		IMU		Population size	Students participated
	
	N	p	n	p	n	p		
1	160	50	100	25	140	50	400	125
2	135	50	50	25	124	50	309	125
3	100	50	50	25	125	50	275	125
4	102	50	51	25	106	50	259	125
**Total**	497	200	251	100	495	200	1243	500

n: estimated population size, p: number of students participated

### Statistical analysis

Both descriptive and inferential data analyses were carried out using Statistical Package for the Social Sciences, SPSS^® ^version 18 with 0.05 as the level of significance. Descriptive statistics were used to analyze frequency, percentage and mean. Chi-square test was performed to measure the association between the demographic characteristics and responses to understanding, perceptions and self-use of CAM. The variables analyzed using Spearman test was similar to that analyzed using Chi-square test. Kruskal-Wallis test was used to analyze the overall differences in terms of their understanding of CAM, from first to fourth year. However, to analyze the difference between two variables such as two different professional years, Mann-Whitney was used.

## Results

### Demographic characteristics

Almost three quarters (77%) of the participants were female with an average age of 21.4 years. Chinese and Buddhist students constituted the highest proportion of 58% and 41.4% students respectively. The number of participants were same, 125 (25%) each from year one to year four respectively. The demographic characteristics of participating students are summarized in Table 3.

**Table 3:** Demographic characteristics of study participants (n = 500)

Variables	n (%)
**Gender, No (%)**	
Male	116 (23.2)
Female	384 (76.8)
**Age, y**	21.44
**Ethnicity, No (%)**	
Malay	159 (31.8)
Chinese	290 (58.0)
Indian	46 (9.2)
Others	5 (1.0)
**Year of Study, No (%)**	
First	125 (25.0)
Second	125 (25.0)
Third	125 (25.0)
Fourth	125 (25.0)
**Religion, No (%)**	
Islam	160 (32.0)
Buddhist	207 (41.4)
Christian	76 (15.2)
Hindu	40 (8.0)
Free thinker	3 (0.6)
Taoism	2 (0.4)
Sikh	1 (0.2)
Baha'i	1 (0.2)
**Type of University, No (%)**	
Public	151 (30.2)
Private	349 (69.8)

### Sources of information for CAM

The Internet was found to be the most commonly used source of information on CAM (69%) from the eight options provided in the questionnaire, followed by friends or family members (63%) and media (61%) which included television, radio and newspaper. Interestingly half of the students acquired information on CAM from CAM practitioners (53%) and healthcare providers (51%) compared to 27.4% of students who obtained information on CAM from their formal education.

### Self-use of CAM among students

More than half (58%) of the participated students were currently using at least one type of CAM while 78% had used it previously. Among those currently using CAM, CM was most commonly used (21.9%) followed by TCM (21%) such as Chinese herbs (42%) and ginseng (22.6%). Previously 35% and 22% of the participants had used ginseng and Chinese herbs. Almost half of the participants stated that they may recommend TCM to their patients, friends and family while 35% asserted that they would recommend TCM as pharmacists. Three most common types of CM used by the students at the time of the study were music and art therapy (36.4%), prayer healing (29.5%) and meditation (25.0%). The self-use of CAM among study participants is presented in Table 4.

**Table 4:** Self-use of CAM among study participants

Modalities	Frequency, n (%)			% Change
		
	Current Use		Previous Use	
Traditional Malay Medicine	45 (9.7)		89 (19.5)	9.8
Traditional Chinese Medicine	105 (21.0)		219 (47.3)	26.3
Traditional Indian Medicine	10 (2.2)		31 (6.9)	4.7
Homeopathy	22 (4.8)		49 (10.9)	6.1
Complementary Medicine	107 (21.9)		187 (39.3)	17.4

**Self-use of CAM modalities (current use)**				

**Modalities**	**Year of study**	**CAM use** **Yes/No**	**Chi-square**	**Correlation**

Traditional Malay Medicine	1 2 3 4	13/80 10/112 13/103 9/126	p = 0.269	Rs = 0.067 p = 0.147
Traditional Chinese Medicine	1 2 3 4	17/80 30/93 23/93 35/106	p = 0.473	Rs = -0.044 p = 0.337
Traditional Indian Medicine	1 2 3 4	0/92 3/117 6/107 1/132	p = 0.023	Rs = -0.022 p = 0.625
Complementary medicine	1 2 3 4	14/84 27/97 35/86 31/114	p = 0.073	Rs = -0.061 p = 0.181
Homeopathy	1 2 3 4	1/91 8/112 8/105 5/125	p = 0.099	Rs = -0.026 p = 0.574

**Figure 1:**
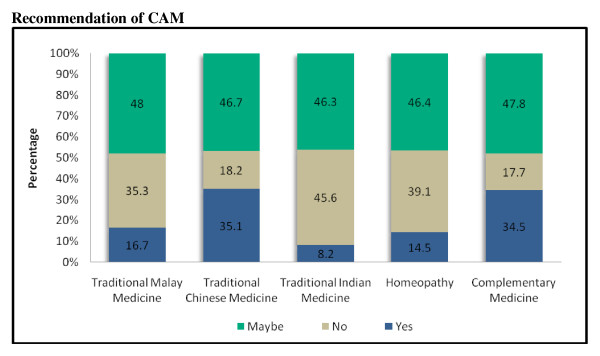
**Responses to questions on recommendation of CAM to patients, friends and family**.

A small percentage (9.7%) were currently using TMM such as *Malay herbs, minyak panas (medicated hot oil), ubat gamat (medicated ointment, it is the end products of sea cucumber/plant), bekam (blood cleaning procedure/used when one person believed that the patient's blood is toxic or dirty), jamu (herb derived from different types of shrub) and misai kucing (Clerodendranthus spicatus/believed to be able to treat diabetes mellitus) *while 89 (19.5%) students had used TMM previously. In this study, homeopathy was found to be more commonly used than TIM. About 15% of the participants revealed that they would recommend homeopathy as pharmacists. However both homeopathy and TIM were used less frequently (6.1% and 4.7% respectively) compared to previous use. The students' responses to questions on recommendation of CAM are presented in Figure 1.

### Understanding of CAM

More than half of the participants (n = 264, 52.8%) agreed that the physical and mental health is maintained by an underlying energy or vital force (mean value 4.66). This statement was significantly and positively correlated with year of study (R_s _= 0.151, p = 0.001) where more senior students (3^rd ^and 4^th ^year) inclined to agree with the statement than junior students (1^st ^and 2^nd ^year). Most of the participants responded positively to the following statements: health and disease is a reflection of balance between positive life-enhancing forces and negative destructive forces (mean score = 4.87); the body is essentially self-healing and the task of a healthcare provider is to assist in the healing process (mean score = 5.13). Conversely, regarding the question describing complementary therapies as a threat to public health, most of the participants had a neutral view (mean value = 3.65). About 60% of students seemed to agree that all treatments that are not tested in a scientifically recognized manner should be discouraged (mean score = 4.79). On the other hand, students also indicated that CAM included beliefs and methods from which conventional medicine could benefit (mean score = 4.71) and was significantly positively correlated to their year of study (R_s _= 0.125, p = 0.005). Post-hoc analysis with Mann-Whitney test showed significant differences between students from first-year and second, third and final years students when compared for responses to the statements assessing understanding of CAM. The results of analyses are summarized in Table 5.

**Table 5:** Overall differences and differences in mean score between each year of study and questions assessing understanding on CAM

Statements	Year of Study (Mean)				Overall difference	Differences between Years of Study					
			
	1	2	3	4		1-2	1-3	1-4	2-3	2-4	3-4
1. The physical and mental health is maintained by an underlying energy or vital force	4.31	4.77	4.61	4.84	0.001	0.005	0.037	0.001	0.524	0.271	0.095
2. Health and disease are a reflection of balance between positive life-enhancing forces and negative destructive forces	4.42	4.94	4.84	5.14	0.001	0.004	0.006	0.001	0.937	0.084	0.109
3. The body is essentially self-healing and the task of a health care provider is to assist in the healing process	4.79	5.26	5.23	5.15	0.016	0.005	0.007	0.026	0.941	0.391	0.372
4. A patient's symptoms should be regarded as a manifestation of general imbalance or dysfunction affecting the whole body	4.47	4.95	4.97	5.12	0.001	0.001	0.001	0.001	0.674	0.142	0.323
5. A patient's expectations, health beliefs and values should be integrated into the patient care process	4.83	5.16	5.08	5.25	0.035	0.042	0.077	0.003	0.683	0.456	0.262
6. Complementary therapies are a threat to public health	3.82	3.73	3.51	3.57	0.266	0.065	0.081	0.136	0.209	0.309	0.776
7. Treatments not tested in a scientifically recognized manner should be discouraged	4.61	4.82	4.88	4.82	0.501	0.212	0.132	0.292	0.887	0.900	0.710
8. Effects of complementary therapies are usually the results of a placebo effect	4.01	4.35	3.92	3.92	0.016	0.017	0.668	0.662	0.009	0.006	0.975
9. Complementary therapies include ideas and methods from which conventional medicine could benefit	4.36	4.87	4.75	4.77	0.001	0.001	0.003	0.001	0.397	0.704	0.633
10. Most complementary therapies stimulate the body's natural therapeutic powers	4.25	4.91	4.74	4.67	0.001	0.001	0.001	0.001	0.458	0.137	0.467

### Perception towards CAM use

Most of the participants reported not knowing about the effectiveness of most of the types of CAM; exceptions to this were TCM (62.6%; mean score = 2.32) and CM such as music and art therapy (53%; mean score = 2.41) and massage (71%; mean score = 2.19). Some of the participants (22, 4.4%) perceived hypnosis as harmful as compared to other types of CAM. TIM revealed to have significant association (p = 0.001) and negative correlation (R_s _= - 0.321) with ethnic group where Indians were agreed to believe that TIM is effective. Similarly, Malays and Chinese tend to perceive that TMM and TCM to be effective. Among CM, prayer healing or faith healing was significantly associated and positively correlated with race where Malays seemed to agree that prayer healing is effective. Significant negative correlations were found with music and art therapy and race, where Chinese and Indians perceived music and art therapy to be effective compared to Malays. The associations of perception towards CAM with demographics are presented in Table 6.

**Table 6:** Association of perceptions towards CAM with demographic characteristics of the pharmacy students

Modalities	Means	Chi-square (p-value)				
		
		Gender	Age	Race	Year of Study	Type of University
Traditional Malay medicine	2.74	0.009	0.005	0.001	0.006	0.001
Traditional Chinese medicine	2.32	0.543	0.597	0.001	0.146	0.001
Traditional Indian medicine	2.84	0.890	0.776	0.001	0.004	0.016
Homeopathy	2.73	0.012	0.226	0.001	0.424	0.001
Complementary Medicine						
*Faith healing/prayer healing*	2.47	0.281	0.161	0.001	0.031	0.001
*Meditation*	2.45	0.090	0.632	0.001	0.018	0.019
*Visualization*	2.79	0.004	0.031	0.039	0.384	0.480
*Hypnosis*	2.77	0.131	0.016	0.001	0.468	0.194
*Music and art therapy*	2.41	0.038	0.222	0.001	0.072	0.001
*Mind-body technique*	2.60	0.010	0.045	0.020	0.258	0.613
*Massage*	2.19	0.993	0.232	0.164	0.678	0.022
*Therapeutic Touch*	2.66	0.077	0.136	0.001	0.281	0.115

*1 = very effective, 2 = Effective, 3 = No idea, 4 = Harmful, 5 = Very Harmful

### Barriers to the use of CAM

**Figure 2:**
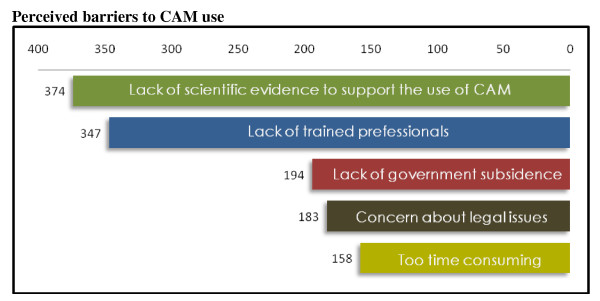
**Responses in numbers for perceived barriers to the use of CAM**.

The majority of the participants claimed that the main barriers to the use of CAM were insufficient scientific evidence to support CAM use (374, 75%) and lack of trained professionals of CAM (347, 69.4%). Interestingly, 39% of the participants thought that a barrier was caused by lack of government financial support for CAM. More than one third were (37%) concerned about the legal issues of the use of CAM and 32% thought that it is too time consuming to use CAM. The list of perceived barriers to the use of CAM is presented in Figure 2.

### Integration of CAM

**Figure 3:**
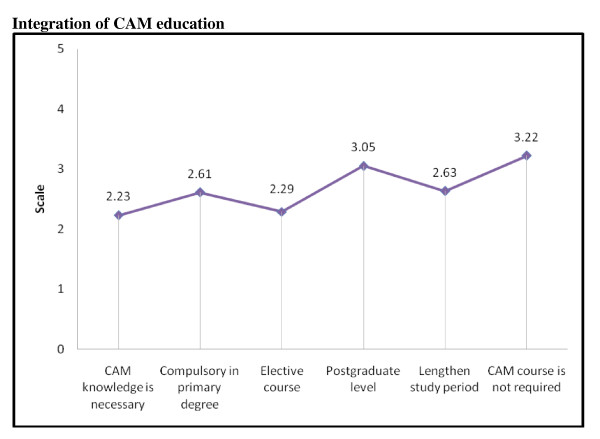
**Responses to questions on integration of CAM education (Scale: 1 = strongly agree to 5 = strongly disagree)**.

Majority of the students (69.0%, mean score = 2.33) agreed that CAM knowledge is necessary to be a well-rounded professional with significant association with race (p = 0.003) and year of study (p = 0.010) where more Malays and senior students agreed to this statement. More than half of them (66%) agreed that CAM should be offered as an elective course and not a compulsory course. Nearly half (47%) felt that latter would lengthen the study period. Regarding the statement that 'CAM course is not required at all as it is the job of CAM practitioner', about 41% of the students disagreed while 39% were neutral (mean score of 3.22). The responses to questions on integration of CAM education in the pharmacy curriculum are presented in Figure 3.

## Discussion

The participating pharmacy students in the public and private universities of Malaysia had, at the time of the investigation, a relatively high level of understanding and positive perception toward most aspects of CAM. Current self-use of CAM among the students was less than previous use. In Malaysia, the majority of the population is Malay (~54%) followed by Chinese (~ 25%) and Indian (~8%). However, this trend was not reflected in the study sample where the majority of the students were Chinese (58%). This is attributed to the participation of two private universities where the majority of students were Chinese. About one quarter of the students agreed that CMs are a threat to public health which is in contrast to a study in Australia where less than 5% of pharmacy students agreed with this statement [6]. Respondents remained neutral when asked whether CMs caused a placebo effect. These two phenomena, in combination, reflect that pharmacy students in Malaysia had more skeptical attitudes towards CAM than students in the US, the UK and Australia [6,7,9,10]. This could be attributed to the limited available evidence-based information regarding CAM in Malaysia compared to information that is not evidence-based.

More than half of the pharmacy students participating in this study thought that CAM includes beliefs and methods from which conventional medicine could benefit. This finding was lower than that reported in Australia and the US [6,10,13]. Interestingly, studies in Singapore and the US documented a higher percentage of medical students who agreed with this statement [15-17]. The response to this statement was significantly different among first year students and those from second year onwards; first year students tend to disagree that CAM includes ideas and methods from which conventional medicine could benefit. The teaching of the CAM module which starts, from the second year could be one of the possible factors contributed to this finding.

Similar to the previous studies, the students in this study perceived lack of scientific evidence as the greatest barrier for them to use CAM [6,10,16]. In addition, the students ranked 'lack of trained professionals' as the second potential barrier, followed by 'lack of government subsidy'. This ranking order was similar to that reported in other studies [6,10]. However, the majority of students recognized the need for CAM knowledge as a well-rounded professional. This could explain why most of the students thought that trained professionals play an important role in convincing them to use CAM.

About 78% reported to have used at least one type of CAM in the past while 58% of them were using CAM at the time of survey. This percentage is higher than for pharmacy students in the US [13], the UK [9], and Hong Kong [8]. More or less equal to that of medical students in the US [17,18], and lower than the percentage of pharmacy students reported in Australia [6]. However this study found that overall the current CAM use was reduced compared to previous use.

Almost half of the participants reported to have used TCM previously. This finding is consistent with that found in Hong Kong [8]. The high percentage of TCM use seems to be related to the number of participating Chinese students (58%) and they perceived TCM as more effective than those of other races in this study. Interestingly, it was also found that the perceived impact and self-use of TCM were significantly associated with race. Family could be one of the factors that influence the students' use of TCM since family members were reported to be a (highly ranked) source from where students acquired their CAM information. This is supported by an Australian study which reported that the attitudes of the students towards CAM were strongly influenced by family [6]. In the UK, one quarter of the students from different ethnic groups claimed to have used TCM, mainly ginseng and ginkgo [13]. Similarly, this study also reveals that the type of TCM which was most frequently used by the students was ginseng. The increasing number of clinical trials that investigated ginseng may have contributed to its expanded popularity [19].

Prayer healing, meditation, massage and music and art therapy were among the most commonly used CMs reported by the participating students. In the US, significant proportions of pharmacy and medical students were reported to have used these four types of CM [15-18]. However, a higher percentage of pharmacy students in this study perceived massage and music therapy to be effective compared to that reported in the US [10]. This difference could be due to the wider variety and availability of traditional massage in Asian countries namely *Malay urutan (Malay Massage), Thai massage*, *Chinese Tui Na massage *(*Tui Na is an Oriental Bodywork Therapy*) and others. This study revealed no differences in CM use among students from different years of study. On the other hand, in contrast to the study in the UK which reported aromatherapy and acupuncture to be most frequently used [9], none of the students reported using aromatherapy while only one student claimed to have used acupuncture previously. The CAM modalities listed in the questionnaire in this study were different to those in the UK study which listed a total of ten types of CAM without specific categories.

Surprisingly, homeopathy reported was found to be used more than TIM. A higher percentage of students claimed that they would recommend homeopathy to their friends, family members or patients compared to those who would recommend TIM. These findings imply that the popularity of the TIM is not as pronounced as homeopathy. The history of homeopathy in the Malay community began in the 1930s and could be the possible reason behind this phenomenon [20]. Moreover, it has been reported that almost half of the students in Malaysia had consulted homeopathic practitioners in addition to conventional treatment [21]. The current study reveals that the acceptance of homeopathy among students has grown more than TIM.

### Limitations of the study

This study had several limitations that may affect its generalization. There were some limitations identified in this study pertaining to its design which did not allow a baseline assessment of specific type of CAM. There were large numbers of CAM used by the students which makes it difficult to concentrate on specific type of CAM. Since data was collected by the lecturers, potential desirable responses in a student-teacher relationship were possible. Self-reported information about CAM use did not rule out selection bias, the possibility of socially desirable responses, and probable variation in data collection, and hence may not reflect the actual behavior and/or practice of a student. Future studies may reveal specific type of CAM practice. A comprehensive comparison with other studies was difficult owing to the unique characteristics of each population, different healthcare systems, methodologies used and sample size.

## Conclusions

This study reveals a relatively high and noticeable percentage of pharmacy students who were using (57.8%), or had previously used (77.8%), at least one type of CAM. Significant differences were found between students from different professional years in terms of their understanding about CAM, and students from higher year of study tended to agree that CMs includes beliefs and methods from which conventional medicine could benefit. Compared to Chinese students, Malay students were more likely to believe that CAM is a threat to public health. However, as the racial breakdown of the participating students did not correspond to the racial breakdown of Malaysia, the results of this study may not be representative of all the pharmacy students in the country. Integration of CAM education into curriculum gives students a chance to provide accurate and unbiased information on CAM in future as pharmacists and to work collaboratively with CAM users to develop treatment plans that optimize adherence to prescribed treatments, satisfaction with care, and health outcomes.

## Competing interests

The authors declare that they have no competing interests.

## Authors' contributions

SSH, CSY and MGB participated in concept, design, data collection, data analysis, data interpretation and writing. SMI, AH and MRB participated in data analysis, data interpretation and writing. CMN and TK participated in concept, design, and writing. All authors read and approved the final manuscript.

## Pre-publication history

The pre-publication history for this paper can be accessed here:

http://www.biomedcentral.com/1472-6882/11/95/prepub
